# Development and validation of an instrument to measure the Micro-Learning Environment of Students (MLEM)

**DOI:** 10.1186/s12909-023-04381-3

**Published:** 2023-05-31

**Authors:** Zainab Akbar, Rehan Ahmed Khan, Humaira Fayyaz Khan, Rahila Yasmeen

**Affiliations:** 1 Rehman College Of Dentistry, Peshawar, Pakistan; 2 Riphah International University, Islamabad, Pakistan

**Keywords:** Environment, Learning, Teaching, Reliability, Validity

## Abstract

**Background:**

There are multiple instruments to measure different learning environments but no valid and reliable instrument is present for measuring the micro-learning environment. This study aimed to develop and validate an instrument to measure the micro-learning environment of students. Measuring the micro-learning environment can give insight into the real-life experiences of students and enlighten us about the disparity between taught, delivered, and learned curricula.

**Methods:**

Multi-institutional Mixed methods study design with consecutive qualitative and, quantitative components was used based on information processing theory. Literature review, results of semi-structured interviews, and focus group discussion were combined to develop a questionnaire. Content and response process validity were established followed by pilot testing, reliability calculation, and exploratory and confirmatory factor analysis.

**Results:**

A forty-nine-item preliminary draft instrument was reduced to a total of twenty-four items final instrument having five themes regarding teaching practices, learners support, competence in teaching, progressive faculty, and teaching environment. The values of SCVI/Ave and S-CVI/UA were calculated to be 0.92 and 0.62 respectively. Reliability was calculated to be 0.94. Fit indices values were within the normal range.

**Conclusion:**

The instrument for measuring the micro-learning environment has excellent content, construct, response process validity, and reliability.

**Supplementary Information:**

The online version contains supplementary material available at 10.1186/s12909-023-04381-3.

## Background

Linder defined micro-learning as a new learning system based on micro content and micro media in the new media ecosystem [1]. It is also termed as a chunk-sized or bite-sized learning [2]. Chunking of the large contents into small pieces, the flexibility of learning time and space, and the availability of multiple learning media choices to learn selectively are all salient features of micro-learning [1]. Existing tools like the Dundee Ready Educational Environment Measure (DREEM), Post-graduate educational atmosphere measure(PHEEM), Anesthetic Theatre Educational Environment Measure (ATEEM), and Surgical Theatre Educational Environment Measure(STEEM) are designed for a specific group of the population only. They do not cater to all healthcare professionals. Besides, the existing tools are quite lengthy(50 items in DREEM, 40 in PHEEM, 42 ITEMS IN CLE, and 50 ITEMS IN AMEET). They are not suitable for short placements [3]. Thirdly, views of all three important stakeholders of the learning environment like students, teachers, and medical education experts are sought on the issue. Previous tools considered only one stakeholder. This makes this tool more comprehensive, up-to-date, and practical to use [4]. The purpose of our study is to develop and validate an instrument to measure the microlearning environment of students(Microlearning Environment measure-MLEM). Measuring the micro-learning environment can give insight into the real-life experiences of students, and enlighten us about the disparity between taught, delivered, and learned curriculum.

Microlearning can either be technology or non-technology based. In Pakistan, participants of our study experienced technology-based microlearning in the form of gamification, video podcasting, e-learning, and microblogs such as Twitter, Whatsapp, Facebook, etc. Besides that, Nontechnological tools of microlearning that are commonly practiced by Pakistani students include texts (phrases, short paragraphs), Images, Videos, Audio, Tests, and Quizzes. Conventional classroom teaching has become monotonous due to the latest wave in digitalization and the introduction of innovative techniques like learning through gamification etc [5]. Including technology-based microlearning strategies can reduce learning time, increase academic performance and improve knowledge or skills [6, 7]. Besides, traditional classroom education has constraints of time and space. Mobile learning and video podcasting are promising tools for medical education [6]. These sources can be accessed anywhere and anytime [8, 9]. Specific use of microblogs such as Twitter can enhance students’ engagement and performance [10]. The term microlearning is relatively new in medical education and all its characteristics, components, target population, and modalities need to be unraveled in-depth to use it effectively as a novel teaching and learning strategy. An emerging trend of digitalization has reduced our concentration span to the extent that engaging and vibrant microlearning environments are the need of the hour.

## Methods

### Design

**Fig. 1:**
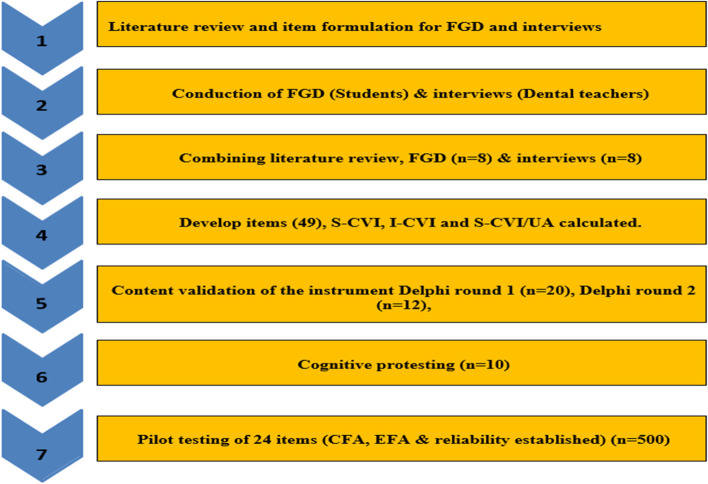
Flow chart of Phases of development of the instrument (MLES)

Mixed method study design, having Consecutive qualitative and quantitative components (Exploratory sequential design), was selected. The instrument development process followed seven steps of AMEE guide 87 [11](Fig. 1).

### Setting

The study involved multiple institutes. It included expert medical educationists, students, and medical faculty teaching in public, private, and military medical colleges, and teaching hospitals in Pakistan. The tentative duration of the study was eight months, once the synopsis is approved, i.e., from Feb 2021 to October 2021. The research was conducted after approval from the ethical review committee of Islamic International Medical College, Reference no. Appl. # Riphah /IRC/ 21/28, and following the Declaration Of Helsinki. Ethical approval and institutional permission were obtained from Rehman college of dentistry (focus group discussions and interviews were conducted) and other dental colleges as well (where the survey was conducted). A brief description of the research was shared with all participants and informed consent was taken before all three phases. Participants’ identity was kept anonymous.

Broadly our study can be divided into three phases.

#### Phase 1: instrument design and construction

After a literature search and critical analysis, qualsyst criteria were applied for shortlisting relevant articles. Themes pertinent to the current study were identified and focus group discussions(FGD) and interviews were planned according to that [12]. Two FGDs were conducted at Rehman college of dentistry, Peshawar. Eight final-year dental undergraduates were selected through a purposive sampling technique. Students were selected for focus group discussion as they are one of the prime stakeholders of any learning environment so they can converse at length and in-depth regarding this topic. The principal researcher took the responsibility of moderator while one of the members of the team was given the responsibility of timekeeping and the other was assigned the role of a scribe. After taking informed consent from the participants, a brief introduction of the research project was given through power point presentation. Discussion among eight group participants was generated based on open-ended questions. Concurrent data analysis paved the way forward for formulating interview questions.

Semi-structured interviews were conducted to develop an in-depth understanding of the perceptions of teachers regarding the microlearning environment. Teachers of both clinical and nonclinical departments who were willing to participate and were having teaching experience of at least five years were selected through a purposive sampling technique. A sample size of eight was achieved based on theoretical data saturation, pragmatic considerations (literature-based), information-rich data, and sample size guidelines. A guided interview protocol was employed. Questions were asked followed by probing questions and comments. Interview questions were refined through piloting and expert validation. Each interviewee was provided with a brief project summary and a written consent form was signed before approximately thirty minutes face to face interviews. Interviews were conducted at different departments at per participant’s convenience. The principal investigator conducted all interviews herself and audio-recorded them. Simultaneous data analysis was done to amend the data collection process and include the emerging themes in succeeding interviews. The final instrument consisted of forty-nine items. Instead of using the format of the questions, items were written in statements. Four-point Likert scale was used for closed-ended survey questions.

#### Phase 2: instrument validation

A modified Delphi technique having two rounds was employed. The Delphi techniques have been used by investigators for ages, both in classical and modified forms, for validation of questionnaires [13, 14].

There is no mutual agreement regarding the number of experts for expert validation. Literature suggests that though ten to hundred experts can be contacted, for clearer consensus regarding clarity of construct; usually up to twenty to thirty experts are required [11].

**Fig. 2:**
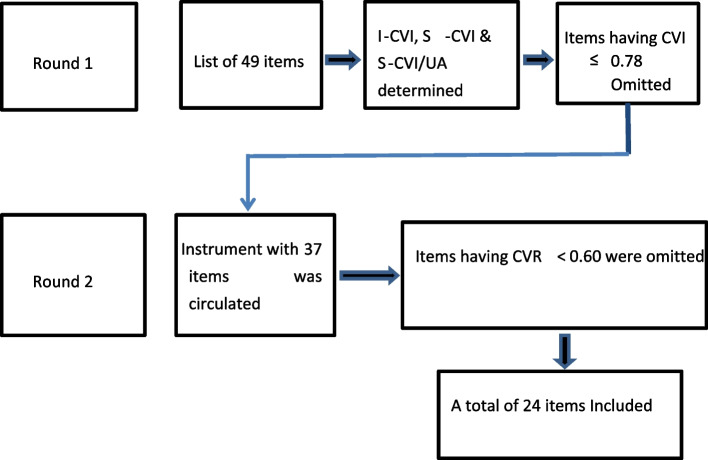
Flow chart of Delphi rounds

A panel of twenty-five medical educationists was selected as experts using purposive sampling techniques from Pakistan and abroad. The participants were sent invitation mail along with the project summary beforehand. Only twenty experts agreed to fill out the questionnaires. Medical educationists that had a Master’s degree in medical education and teaching experience in a medical or dental college of more than five years were included in the study, considering their same subject matter understanding. Four-point Likert scale was provided to participants, and they were instructed to rate items according to relevance to the construct( Highly relevant (HR) 04, Quite relevant (QR)03, Somehow relevant (SR)02, Not relevant (NR)01). Besides that, participants were requested to add their valuable comments or suggestions in the comment box. Participants were encouraged to justify in case they select extremes of options. An open-ended question encouraging panelists to suggest any added comment was included at the end of every section of the questionnaire. Items having CVI of ≥ 0.90 were included, between 0.78–0.90 revised, and Items with I-CVI ≤ 0.78 were removed [13]. The instrument with thirty-seven items was sent for the second Delphi round after the required amendments as suggested by participants. The revised questionnaire with the list of statements that did not reach a consensus from round 1 was mailed to those twenty experts who responded in the first round. During the second round, responses from only twelve experts were received through Google forms. Each item was marked on a four-point Likert scale, considering how essential the item is. The content validity ratio (CVR) was calculated through Lawshe’s formula that is, CVR = (Ne-N/2)/(N/2) (LAWSHE, 1975). Likert scale was used again(Highly essential—4, Quite essential—3, Somewhat essential—2, Not essential—1). A critical value of CVR, for a panel of twelve experts, for retention of an item is 0.6 so items having CVR greater than 0.8 were retained as such, items falling in the range 0.60 to 0.80 were revised and those having values less than 0.60 were omitted [15](Fig. 2).

Cognitive pretesting is used to determine response process validity. Though ten to thirty participants are required for cognitive pretesting, The literature suggests that for small-scale studies, a sample size of five to six may be enough [11]. In our case, a sample size of ten bachelors of dental surgery students, from different private and government dental institutes in Pakistan, were selected for approximately fifteen minutes of cognitive interviews through Non-Probability Convenience Sampling. Interviews were held using the verbal probing method [14].

#### Phase 3: pilot testing and factor analysis

Taking a population sample size of 1200 dental students from six dental colleges (colleges having 50 BDS Students in one class), the sample size was calculated to be 292 by open epi calculator. Simple Random Sampling was utilized. Undergraduate dentistry students from three colleges of Khyber Pakhtunkhwa (Rehman College of Dentistry, Khyber College of Dentistry, Sardar Begum) and Punjab (Army Medical College, Foundation University College of Dentistry, Watim Medical, and Dental college)were selected. To avoid any bias in the study the selection of the participants will be done purely voluntarily and the confidentiality of the participants will be strictly maintained. Five hundred participants were selected for pilot testing and factor analysis through nonprobability purposive sampling. A Questionnaire was distributed to Google forms for online correspondence.

Exploratory factor analysis was done by using SPSS. For Confirmatory factor analysis (CFA), AMOS software was used and for Internal consistency, Cronbach’s alpha was calculated using SPSS version 24.

## Results

### Phase 1: instrument design and construction

The focus group discussions were transcribed manually using THE cycle of coding. In the 1^st^ cycle of coding, meaningful units were identified by the principal investigator. These units were summarized to develop codes. Participants of the focus group categorized 48 meaningful codes. 32 open codes were identified after analyzing the data from interviews. Codes of both focus group discussions and interviews were combined. Codes were again evaluated and similar codes were merged there achieving a final set of five major categories. These categories were considered to be important domains of teaching quality in a microlearning environment.

### Phase 2:instrument validation

Twelve items were removed having I-CVI ≤ 0.78. 16 items were revised based on the value of I-CVI and qualitative feedback from the experts. The remaining 21 items were retained as such. The values of the scale content validity index/ average (S-CVI/Ave) was 0.92 and the scale content validity index/ universal agreement (S-CVI/UA) was 0.61.

For the second round, thirty-seven items were transferred to Google form and circulated to twenty experts (respondents of round one). Based on the responses of twelve participants, the content validity ratio (CVR) was determined. 14 items with a CVR of less than 0.60 were removed. 13 items with a CVR between 0.60–0.80 were revised. Items with a CVR of more than 0.80 were included in the instrument for the next round.

In cognitive pretesting, ten medical teachers participated. It explored the views of participants regarding the statements of the items of the instrument.

### Phase 3:pilot testing and factor analysis

The whole sample size for this study was (n = 500), which was divided into two sub-samples (A and B) equally random groups. First of all, the factor structure was measured by EFA using subsample A (n = 250). Using SPSS 21.0, Exploratory Factor Analysis (EFA) was performed to look for common factors in the latent variables (Table 1). The principal component factoring method was chosen. The goal of this research was to look at the notion of teaching methodologies, training, practices, etc. Using this method, several factors to be extracted were determined to enable this process for meaningful results. The Promax method was used to discover the factors.

**Table 1:** Communalities in exploratory factor analysis

Communalities		
	**Initial**	**Extraction**
1. Teachers are well-organized and follow the schedule	1.000	.665
2. Teachers are well-prepared for every session	1.000	.753
3. Learning objectives are mentioned for each session	1.000	.476
4. Teachers use metacognitive strategies (clues, hints, schemas, examples, questions) during the course delivery	1.000	.543
5. Teachers have effective communication skills	1.000	.591
6. Students are the center of attention in every learning environment	1.000	.423
7. Teachers value my feedback on the course and teaching activities	1.000	.399
8. Teachers encourage me to be an active and lifelong learner	1.000	.547
9. Teachers provide me appropriate guidance required for my learning	1.000	.609
10. Teachers guide me about learning resources for every session	1.000	.479
11. Teachers help me build new knowledge on my prior knowledge	1.000	.561
12. Teachers improve my knowledge, skills, and attitude with their teaching	1.000	.572
13. Teachers provide me with timely and constructive feedback on my learning	1.000	.447
14. Tasks and activities assigned to me correspond with my level of learning	1.000	.459
15. Teachers, being role models, are a source of inspiration for me	1.000	.615
16. Teachers have command over their subject/discipline	1.000	.626
17. Teachers help me in applying theoretical knowledge to practice	1.000	.609
18. Teachers are well-equipped with Online teaching and assessment techniques	1.000	.764
19. Teachers use multiple and innovative teaching methods	1.000	.784
20. Teachers emphasize critical thinking rather than rote memorization	1.000	.692
21. Teachers encourage a friendly learning environment	1.000	.551
22. Teachers have an unbiased approach toward all students	1.000	.954
23. Teachers mock and ridicule the students	1.000	.884
24. Teachers are interested in completing the course instead of clarification of concepts	1.000	.906

Extraction Method: Principal Component Analysis

The loading and cross-loading criterion was set at 0.4, and items with loading less than 0.4 and cross-loading greater than 0.4 were eliminated. This approach was repeated until a simple structure was found in which loading on putative factors was maximized and loading on other factors was minimized. To select a stable, at least three variables per factor were necessary (Table 2).

**Table 2:** Pattern matrix

Pattern Matrix^a^					
	Component				
	1	2	3	4	5
1. Teachers are well-organized and follow the schedule	.822				
2. Teachers are well-prepared for every session	.869				
1. 1. Teachers use metacognitive strategies (clues, hints, schemas, examples, questions) during the course delivery	.730				
2. 2. Teachers have effective communication skills	.754				
8. Teachers encourage me to be an active and lifelong learner		.720			
9. Teachers provide me appropriate guidance required for my learning		.706			
10. Teachers guide me about learning resources for every session		.719			
11. Teachers help me build new knowledge on my prior knowledge		.876			
12. Teachers improve my knowledge, skills, and attitude with their teaching		.634			
14. Tasks and activities assigned to me correspond with my level of learning		.598			
15. Teachers, being role models, are a source of inspiration for me			.711		
16. Teachers have command over their subject/discipline			.790		
17. Teachers help me in applying theoretical knowledge to practice			.808		
18. Teachers are well-equipped with Online teaching and assessment techniques				.979	
19. Teachers use multiple and innovative teaching methods				.940	
20. Teachers emphasize critical thinking rather than rote memorization				.949	
22. Teachers have an unbiased approach toward all students					.874
23. Teachers mock and ridicule students					.821
24. Teachers are interested in completing the course instead of clarification of concepts					.453

Extraction Method: Principal Component Analysis

Rotation Method: Promax with Kaiser Normalization

^a^Rotation converged in 5 iterations

The factorial validity of the scale was using EFA on all items. The Kaiser–Meyer–Olkin sampling adequacy score was 0.804, Chi-square approx. value was 2363.785 and Bartlett’s sphericity test was statistically significant (P = 0.000), indicating that this data was well-suited to factor analysis (Table 3). A five-factor solution with 24 items that accounts for 64.475% of the total item variances in the database was obtained.

**Table 3:** KMO and Bartlett’s Test

KMO and Bartlett's Test		
Kaiser–Meyer–Olkin Measure of Sampling Adequacy		.804
Bartlett's Test of Sphericity	Approx. Chi-Square	2362.785
	Df	171
	Sig	.000

Factor 1 “Teaching practices” with 5 items accounted for 9.945%, Factor 2 “Learners Support with 9 items accounted for (27.491%), Factors 3 “Competence in Teaching” with 3 items accounted for 8.380%, Factor 4 “Progressive Faculty” with 3 items accounted for 5.814% and Factor 5 “Teaching Environments” with 4 items accounted for 13.845% (Table 4).

**Table 4:** Variance in exploratory factor analysis

Total Variance Explained							
**Component**	**Initial Eigenvalues**			**Extraction Sums of Squared Loadings**			**Rotation Sums of Squared Loadings**
	Total	% Of Variance	Cumulative %	Total	% Of Variance	Cumulative %	Total
2	1.889	9.945	51.280	1.889	9.945	51.280	2.625
3	5.223	27.491	27.491	5.223	27.491	27.491	4.351
4	1.592	8.380	59.661	1.592	8.380	59.661	2.303
2	2.631	13.845	41.336	2.631	13.845	41.336	3.659
5	1.105	5.814	65.475	1.105	5.814	65.475	3.009

Extraction Method: Principal Component Analysis

When components are correlated, sums of squared loadings cannot be added to obtain a total variance

**Fig. 3:**
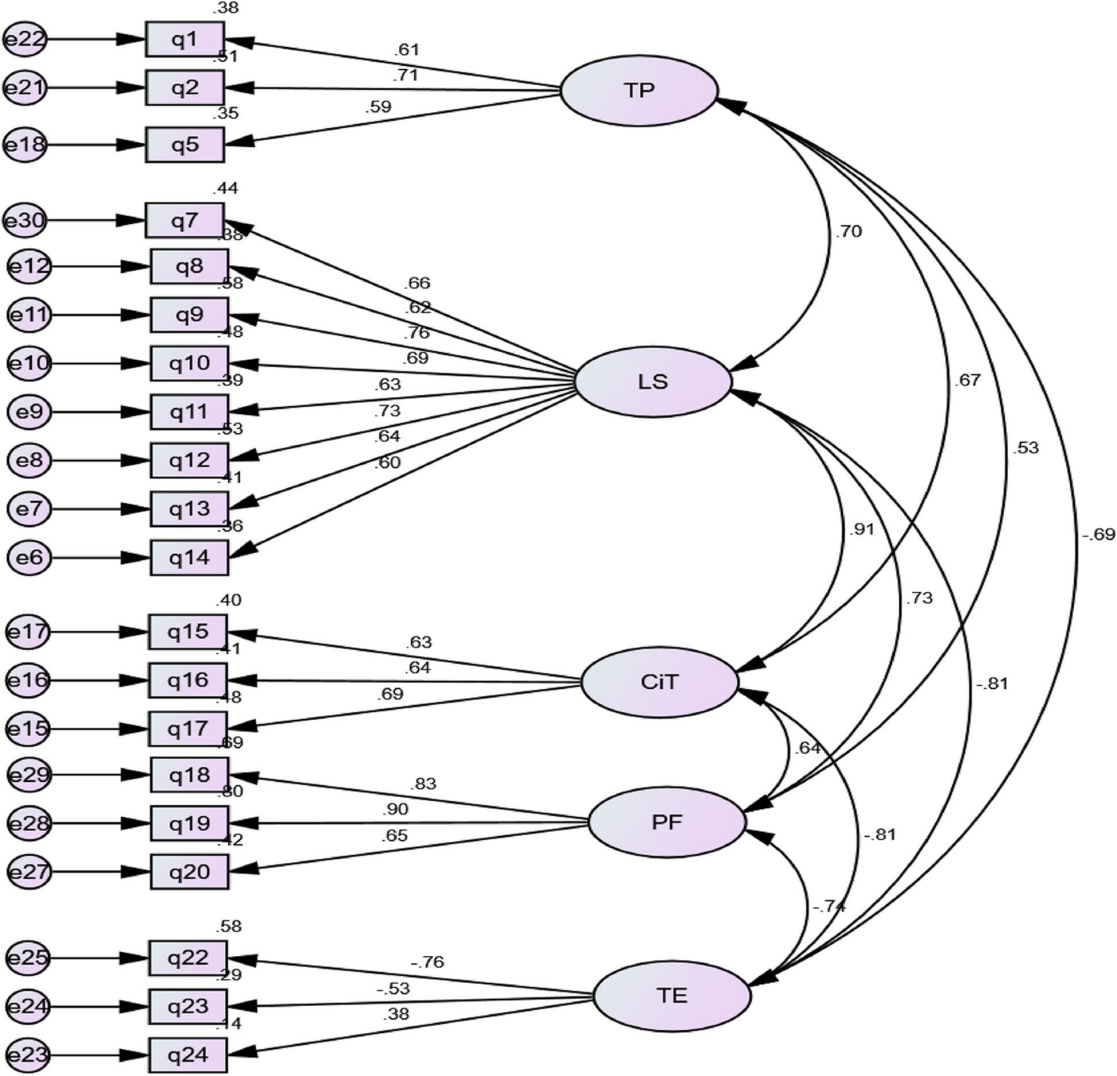
Confirmatory Factor Analysis using AMOS. “TP: Teaching practices” “LS: Learners Support” “CIT: Competence in Teaching” “PF: Progressive Faculty” “TE: Teaching Environments”

The factor structure was cross-validated using Confirmatory Factor Analysis (CFA). The data from the sub-sample B (N = 250) were used for CFA to further confirm the factor structure’s stability. The CFA model’s goodness of fit was evaluated using a variety of model fit indices. The Root mean square error of approximation (RMESEA), Comparative Fit Index (CFI), Standardized root mean square residual (SRMR), Incremental fit index (IFI), and other indices were used in this investigation. The factor loading of regression weights of all the components is above 0.50 except q24. That was retained due to the requirements of each variable (at least 3). The Path model is shown with regression weights (Fig. 3).

A confirmatory factor analysis (CFA) was performed using AMOS 21.0 for the other (*n* = 250) cases, those were randomly selected for CFA and EFA equally and found the results for the same five-factor structure. Many CFA fit indices indicated an excellently good fit for the model. These indices show the confirmatory proof for the factor structure in the following tables (Table 5, 6, 7, 8, 9, and 10).

**Table 5:** CMIN

Model	NPAR	CMIN	DF	P	CMIN/DF
Default model	50	281.181	160	.000	1.757
Saturated model	210	.000	0		
Independence model	20	2146.835	190	.000	11.299

**Table 6:** RMR, GFI

Model	RMR	GFI	AGFI	PGFI
Default model	.053	.900	.869	.686
Saturated model	.000	1.000		
Independence model	.326	.275	.199	.249

**Table 7:** Baseline comparisons

Model	NFI Delta1	RFI rho1	IFI Delta2	TLI rho2	CFI
Default model	.869	.844	.939	.926	.938
Saturated model	1.000		1.000		1.000
Independence model	.000	.000	.000	.000	.000

**Table 8:** RMSEA

Model	RMSEA	LO 90	HI 90	P CLOSE
Default model	.055	.044	.066	.207
Independence model	.203	.196	.211	.000

**Table 9:** Goodness of fit indices

Measure	Estimate	Threshold	Interpretation
CMIN	281.181	–	–
DF	160	–	–
CMIN/DF	1.757	Between 1 and 3	Excellent
CFI	0.938	> 0.95	Acceptable
SRMR	0.060	< 0.08	Excellent
RMSEA	0.055	< 0.06	Excellent
PClose	0.207	> 0.05	Excellent

**Table 10:** Cutoff criteria^a^

Measure	Estimate	Threshold	Interpretation
CMIN/DF	> 5	> 3	> 1
CFI	< 0.90	< 0.95	> 0.95
SRMR	> 0.10	> 0.08	< 0.08
RMSEA	> 0.08	> 0.06	< 0.06
P Close	< 0.01	< 0.05	> 0.05

^a^Hu and Bentler (1999, "Cutoff Criteria for Fit Indexes in Covariance Structure Analysis: Conventional Criteria Versus New Alternatives") recommend combinations of measures. I prefer a combination of CFI > 0.95 and SRMR < 0.08. To further solidify evidence, add the RMSEA < 0.06

Whereas the summary table with its cut values and actual value are as follows for the goodness of fit indices.

## Discussion

Our instrument was developed based on Information processing theory which suggests that working memory can store information in small pieces and can be retrieved through repetition or proper organization (chronology or chunking). AMEE guide No. 87 was utilized in the current study [14]. The themes of our instrument are teaching practices, learners support, competence in teaching, progressive faculty, and learning environment. The reliability of our instrument was found to be 0.94 and individual Cronbach’s alpha for each domain was also more than 0.9 so these can be used independently as well. The content and construct validity of the instrument were also established through Delphi rounds, cognitive pretesting, and confirmatory and exploratory factor analysis.

The concept of a microlearning environment has been identified in the previous literature as well. One of the previous studies related to the development of a brief microlearning environment measure was conducted by Isba et all in 2020. This study was also a mixed method study like ours, but they collected data from healthcare students related to different healthcare professionals. Only literature review and pragmatic data from healthcare students were utilized to develop a questionnaire and only one Delphi round was conducted for expert validation whereas in our study, to get a more holistic view, opinion of the students (focus group discussion), literature review, perceptions of dental teachers(interviews) and later on expert validation (medical educationists) were sought. Two Delphi rounds were conducted to reach to consensus. Teaching quality and staff attitude and behaviors were emphasized only in the HEMLAM instrument while in our case Five domains of teaching quality were explored [16]. Thou developed and validated a new instrument named technology-enabled active learning. inventory (TEAL). It consisted of four scales naming interactive, engagement, problem-solving skills, interest, and feedback. Seven points Likert scale was used for survey purposes. The Moore and Benbasat instrument development process consists of item creation, card sorting, and instrument testing was used to ensure content, construct validity, and reliability. In contrast to that our study followed eight steps of AMEE guide 87 for instrument development. Five-point Likert scale was used to gauge the participants’ responses and our domains revolved only around teaching quality. In addition to that only students’ perceptions are only catered for while in our study. students, teachers, and medical education experts’ views all were recorded and analyzed [17].

A forty-item instrument named E-learning educational atmosphere measure (EEAM) was developed to measure students’ perceptions of E-learning. It covered six domains like program effectiveness, teaching quality, ethics and professionalism, learner support, safety and convenience, and awareness of the rules. Whereas in our study we have assessed different domains of teaching quality in detail. Authors have emphasized the E-Learning environment whereas our study focuses on the microlearning environment [18].

Previous literature by Bruck [19] and Aitchanov, et. al. [20], who used Twitter, a social media technology, Kovachev, Cao, Klamma, & Jarke, (2011) [21] experimented with bilingual vocabulary learning, and Similarly, Wang (2017), investigated the effect of delivering Engineering Mechanic Experiment content in short, sequenced videos all showed promising results with the use of microlearning [22].

The DREEM inventory has 50 items and has five sub-scales relating to Students’ Perceptions of Learning; Students’ Perceptions of Teachers; Students’ Academic Self-Perceptions; Students’ Perceptions of Atmosphere; Students’ Social Self-perceptions. While Our study measures the microlearning environment of undergraduate dentistry students only using inventory having five domains related to teaching quality only [23]. The final 50-item AMEET inventory comprising six domains was used to assess the viewpoints of medical faculty on the educational environment experienced by teachers instead of students [22].

In context to theoretical implication, our study has provided advancement to literature. An emerging trend of digitalization has reduced our concentration span to the extent that engaging and vibrant microlearning environments are the need of the hour. Teachers, students, and healthcare professionals can use our instrument for assessing the micro-learning environment of students at their respective institutes, with a special focus on teaching quality. It can rectify the mistakes in the current system and pave the way forward by increasing student engagement, enhancing student satisfaction, and positively impacting the learning experience.

### Limitations of the study

Though every effort was made to judiciously follow all the steps of instrument development, there are still a few shortcomings that are worth mentioning. Study findings are not generalizable as data is collected from one country only. The primary focus of the study was to get a holistic view of the microlearning environment with the perspective of teaching quality only, other stakeholders like students, administration, and other factors affecting the microlearning environment like easy access to technology, appropriate content formulation and assessment should be taken into account too. The disproportionate number of items across different domains of teaching quality is one of the study's significant shortcomings. To keep the instrument concise, efforts must be made to reduce its size while keeping the factor structure and psychometric qualities intact. The instrument should be validated on a new sample of healthcare students from different professional groups and in different settings countrywide and globally.

### Recommendations for future research

The instrument should be verified in other institutes in Pakistan and abroad to prove its generalizability. The guidelines can help the faculty of colleges to identify flaws in their microlearning environment, providing the basis for perfection in performance. Research can be broadened by taking into account other aspects of the microlearning environment as well. The role of the current instrument in improving the teaching quality after the conduction of workshops and seminars about microlearning environments should be studied in research. To generate a smartphone app using this instrument, that students could use for technology-enabled microlearning and could give instantaneous feedback as well, is a promising idea.

## Conclusion

The final instrument, named MLES, comprised twenty-four items, rated on a four-point Likert scale. The instruments' content validity, construct validity, and reliability was determined. The current study's most notable strength was the appropriate steps of instrument development followed. The administration of different colleges can inculcate this instrument in institute quality evaluation programs to measure the microlearning environment of students, especially from the perspective of teaching quality. The study outcome would provide additional benefits to colleges to improve their micro-learning environments.

## Supplementary Information

**Additional file 1: Appendix I.** FOCUS GROUP DISCUSSION. **Appendix II.** INTERVIEW QUESTIONS. **Appendix III.** Modified Delphi round One. **Appendix IV.** Delphi round Two. **AppendixV.** Results of Cognitive Interviews. **AppendixVI.** QUESTIONAIRE.

## Data Availability

“All data generated or analyzed during this study are included in this published article [and its supplementary information files”. Supporting data is available as ∙ Questions Of Focus Group Discussion (After Expert Validation): Appendix-I ∙ Interview Questions (After Expert Validation) Appendix-II ∙ Delphi Round 1 Questions Appendix-III ∙ Delphi Round 2 Questions Appendix-IV ∙ Results Of Cognitive Interviews. Appendix-V ∙ Questionnaire For Pilot Study Appendix-VI

## Abbreviations

DREEM: Dundee Ready Educational Environment Measure
ATEEM: Anesthetic Theatre Educational Environment Measure
PHEEM: Postgraduate educational atmosphere measure
STEEM: Surgical Theatre Educational Environment Measure
RCD: Rehman College of Dentistry
BDS: Bachelor of dental surgery
FGD: Focus group discussion
EFA: Exploratory factor analysis
CFA: Confirmatory factor analysis
HEMLAM: Healthcare Education Micro-Learning Environment Measure
TEAL: Technology-enabled active learning. Inventory
CVR: Content validity ratio
CVI: Content validity index
Spss: Statistical package of social sciences
RMESEA: The Root mean square error of approximation
CFI: Comparative Fit Index
SRMR: Standardized root mean square residual
IFI: Incremental fit index
