# Supplementary material for: Development and validation of an instrument to measure the Micro-Learning Environment of Students (MLEM)
Source: BMC Med Educ. 2023 May 31;23:395. doi: 10.1186/s12909-023-04381-3 (PMC10233888; doi:10.1186/s12909-023-04381-3)
Supplement: Supplementary file 1 — Additional file 1: Appendix I. FOCUS GROUP DISCUSSION. Appendix II. INTERVIEW QUESTIONS. Appendix III. Modified Delphi round One. Appendix IV. Delphi round Two. AppendixV. Results of Cognitive Interviews. AppendixVI. QUESTIONAIRE. [file 12909_2023_4381_MOESM1_ESM.docx]

**APPENDIX I : FOCUS GROUP DISCUSSION**:

OPENING QUESTION:

Q1. What do you understand by learning environment?

Q2. What makes a learning environment?

Q3. How many types of learning environments are you familiar with?

INTRODUCTORY QUESTION:

Q4. Who, in your opinion, are the main stakeholders of any learning environment?

Q5. What are the main components of any learning environment?

Q6. What is role of teachers in any learning environment?

TRANSITION QUESTION:

Q7. Which teaching characteristics you like the most in your favorite teachers? If yes, then what are those roles?

Q8. How staff attitude affects student knowledge, skills, and behavior?

KEY QUESTIONS:

Q9. What do you think are the ways in which teacher can support a learner?

Q10.Describe what type of learning environment your institution fosters?

Q11. Which in your opinion are the strengths and weaknesses of your learning environment?

Q12.Do you think your teachers are well equipped with teaching and learning strategies? If not, then why? if yes then how?

Q13. How your teachers provide practical insight about theoretical knowledge?

ENDING QUESTION:

Q14.Is there anything that I have not covered so far, or you want to add?

**Appendix II : INTERVIEW QUESTIONS**

**Title Study:** Development and Validation of an Instrument to Measure Micro learning Environment of Undergraduate Dentistry Students

**Researcher:** Dr. Zainab Akbar

**DEMOGRAPHICS:**

A) Designation:

B) Gender:

C) Academic qualification:

D) Years of experience:

| **Teachers**  **interview questions** | | |
| --- | --- | --- |
| 1 | What, in your opinion, is positive and negative learning environment? |  |
| 3 | What are the ways in which a teacher can motivate and support his /her student? |  |
| 4 | Have you brought any change in your learning environment, if yes how, if not why? |  |
| 5. | What new and innovative teaching and learning strategies have you adopted with the passage of time? |  |
| 6. | Are you involved in any faculty development program of your institute in any capacity? if yes then how, if not then why? |  |
| 7. | What reforms do you want to bring in your learning environment for its betterment?    **APPENDIX III : Modified Delphi round One** |  |

| S.NO. | Item | E1 | E2 | E3 | E4 | E5 | E6 | E7 | E8 | E9 | E10 | E11 | E12 | E13 | E14 | E15 | E16 | E17 | E18 | E19 | E20 | Comments | CVI |
| --- | --- | --- | --- | --- | --- | --- | --- | --- | --- | --- | --- | --- | --- | --- | --- | --- | --- | --- | --- | --- | --- | --- | --- |
| 1 | Teachers encourage and promote group activities and interactive discussions. | R | R | NR | R | R | R | R | R | R | R | R | NR | R | R | NR | R | R | R | R | R | Revised | 0.85 |
| 2 | Teachers are well-organized and follow the schedule | R | R | R | R | R | R | R | NR | R | NR | R | NR | R | R | R | R | R | R | R | R | Included | 0.9 |
| 3 | The teachers are well-prepared for every session | R | R | R | R | R | NR | R | R | R | R | R | R | NR | R | R | R | R | R | R | R | Included | 0.9 |
| 4 | Students are the focus of attention in every learning environment | R | R | R | R | R | R | R | R | R | R | R | R | R | R | R | R | R | R | R | R | Included | 1 |
| 5 | Teachers have good time management skills | R | R | NR | R | R | R | R | R | R | R | R | R | R | R | R | R | R | R | R | R | Included | 0.95 |
| 6 | Learning objectives are clearly mentioned for each session. | R | R | R | R | R | R | R | NR | NR | R | R | R | R | R | R | R | R | R | R | R | Included | 0.9 |
| 7 | Teachers provide clear and precise instructions | R | R | R | R | R | R | R | NR | R | R | NR | R | NR | R | R | R | R | R | R | R | Revised | 0.85 |
| 8 | Clinically oriented lectures are delivered | NR | R | R | R | R | R | R | NR | R | NR | R | R | NR | R | NR | R | R | NR | R | R | Removed | 0.7 |
| 9 | Teachers use metacognitive strategies (clues, hints, schemas, examples, questions) during the course delivery | R | R | R | R | R | R | R | NR | NR | R | R | R | NR | R | R | R | R | R | R | R | Revised | 0.85 |
| 10 | Teachers tend to deal patient with a holistic approach, instead of departmentalized. | NR | R | R | NR | R | R | R | NR | R | NR | R | R | NR | R | R | R | NR | NR | R | NR | Removed | 0.65 |
| 11 | Teachers take into account different learning styles of students | R | R | R | R | R | R | R | NR | NR | NR | R | R | NR | NR | NR | NR | R | R | R | R | Removed | 0.65 |
| 12 | Teachers judge their teaching effectiveness by performance of the weakest student of the class. | R | R | NR | R | R | R | R | NR | NR | NR | NR | R | NR | R | NR | R | R | R | NR | NR | Removed | 0.55 |
| 13 | Teachers have effective communication skills | R | R | R | R | R | R | R | NR | R | R | R | R | R | R | R | R | R | R | R | R | Included | 0.95 |
| 14 | Teachers are well versed about rules of power point presentations. | R | R | R | R | NR | R | R | NR | NR | NR | R | R | R | NR | R | R | R | R | 4R | R | Removed | 0.75 |
| 15 | Teachers cover the content in appropriate time during the session | R | R | R | R | R | R | R | NR | R | R | R | R | R | R | R | R | R | NR | R | R | Included | 0.9 |
| 16 | Teachers value my feedback on course and teaching activities | R | R | R | R | R | R | R | NR | R | R | R | R | R | R | R | R | R | R | R | R | Included | 0.95 |
| 17 | Teachers encourage me to be active and lifelong learners. | R | R | R | R | R | R | R | NR | R | NR | R | R | R | R | R | R | NR | R | 4R | R | Revised | 0.8 |
| 18 | Teachers provide me essential guidance suitable for my lea  Rning | R | R | NR | R | R | R | R | NR | R | NR | R | R | R | R | R | R | NR | R | R | R | Included | 0.9 |
| 19 | Teachers provide me essential learning resources after the session. | R | R | NR | R | R | R | R | NR | NR | R | R | R | R | R | R | R | R | R | R | R | Included | 0.9 |
| 20 | Teachers built new knowledge on my prior knowledge | R | R | NR | R | R | R | R | NR | NR | NR | R | R | R | R | R | R | R | R | R | NR | Revised | 0.78 |
| 21 | Teachers improve my knowledge and skills with their teaching. | R | R | R | R | R | R | R | NR | NR | NR | R | R | R | NR | R | R | R | R | R | R | Included | 0.8 |
| 22 | Carrier counselling is being provided by teachers. | R | NR | NR | R | R | R | R | NR | R | R | NR | NR | NR | NR | NR | R | R | R | R | R | Removed | 0.65 |
| 23 | Teachers cover the content in appropriate time during the session | R | R | NR | R | R | R | R | R | NR | N  R | R | NR | R | R |  | R | R | R | R | NR | Revised | 0.78 |
| 24 | Teachers make me feel that they are an important stakeholder | R | R | NR | R | R | R | NR | R | NR | R | NR | R | NR | NR | R | R | R | R | NR | R | Removed | 0.65 |
| 25 | Teachers make sure that required books, instruments and resources are available for me | R | NR | NR | R | R | R | R | NR | NR | NR | R | R | R | NR | NR | R | R | R | NR | R | Removed | 0.6 |
| 26 | I am offered incentives like scholarships if i perform well | R | NR | NR | R | R | R | R | NR | NR | R | R | R | R | NR | R | R | R | R | R | R | Revised | 0.78 |
| 27 | I am never mocked or ridiculed. | R | R | NR | R | R | R | R | R | NR | NR | R | R | R | NR | R | R | R | R | R | R | Revised | 0.8 |
| 28 | Teachers, being positive role models, are a source of inspiration for me. | R | R | R | R | R | R | R | NR | R | NR | R | R | R | R | R | NR | R | R | R | R | Revised | 0.8 |
| 29 | Teachers have optimal teaching skills | R | NR | R | R | R | R | R | NR | NR | R | R | R | R | R | R | R | R | R | R | R | Revised | 0.8 |
| 30 | Teachers’ academic ability and qualification is up-to the mark | R | R | R | R | R | R | R | NR | R | R | R | R | R | R | R | R | R | R | R | R | Retained | 0.95 |
| 31 | Teachers have command over their subject/discipline | R | NR | R | R | R | R | R | NR | R | NR | R | R | R | NR | NR | NR | R | R | NR | R | Revised | 0.8 |
| 32 | Teachers have some degree in medical education, in addition to major specialty. | R | NR | R | R | NR | R | R | NR | R | NR | R | R | R | NR | R | NR | R | R | R | R | Removed | 0.7 |
| 33 | Teachers are well equipped with E teaching and assessment techniques. | R | R | R | R | R | R | R | NR | NR | R | R | R | R | R | R | R | R | NR | R | R | Included | 0.9 |
| 34 | Teachers provide me with timely and constructive feedback on my learning | R | R | R | R | R | R | R | NR | NR | R | R | R | R | R | R | NR | R | R | R | R | Included | 0.9 |
| 35 | Teachers use multiple and innovative teaching strategies | NR | R | R | R | R | R | R | NR | NR | R | R | R | NR | R | NR | R | R | R | R | R | Revised | 0.75 |
| 36 | Teachers demonstrate Mentorship skills in teaching | R | R | R | R | R | R | R | NR | R | R | R | NR | R | R | R | R | R | NR | R | R | Included | 0.9 |
| 37 | Teachers helped me in applying theoretical knowledge into practice. | R | R | R | R | R | R | R | NR | NR | NR | R | R | R | R | R |  | R | R | R | R | Included | 0.9 |
| 38 | Tasks and activities assigned to me corresponded with my level of learning. | R | R | NR | R | R | R | R | NR | NR | R | R | R | R | R | NR | NR | R | R | R | R | Revised | 0.8 |
| 39 | Teachers are receptive to new ideas | R | R | R | R | R | R | R | NR | NR | R | R | R | R | NR | NR | R | NR | R | R | R | Removed | 0.75 |
| 40 | Teachers are flexible in their demeanor. | R | R | NR | R | R | R | R | NR | R | R | R | R | R | R | R | R | R | R | R | R | Removed | 0.65 |
| 41 | Teachers provide me a welcoming and friendly learning environment | NR | R | R | NR | R | R | R | NR | NR | NR | R | NR | NR | R | NR | R | R | R | R | NR | Included | 0.9 |
| 42 | I am involved in decision making processes | R | R | R | NR | R | R | R | NR | NR | NR | R | R | R | NR | NR | R | R | R | R | R | Removed | 0.65 |
| 43 | Teachers Encourage Peer Assisted Learning | R | R | NR | R | R | R | R | NR | NR | R | R | R |  |  | R |  | R | R | R | R | Revised | 0.8 |
| 44 | Teachers promote critical thinking rather than rote memorization | R | R | NR | R | R | R | R | NR | R | R | R | R | R | R | R | R | R | R | R | R | included | 0.95 |
| 45 | Teachers have an unbiased approach towards me. | R | R | NR | R | R | R | R | NR | R | R | R | R | R | NR | R | R | R | NR | R | R | Revised | 0.8 |
| 46 | Teachers mock and ridicule the students | R | R | R | R | R | R | R | R | NR | R | R | R | NR | R | R | R | R | NR | R | R | Revised | 0.8 |
| 47 | Teachers are nonjudgmental towards me | R | R | R | R | R | R | R | NR | NR | R | R | R | R | R | R | R | R | R | R | R | Included | 0.9 |
| 48 | I feel that Teachers are, more interested in finishing the course, instead of learning of students | R | R | R | R | R | R | R | R | R | R | R | R |  |  | R |  | R | R | R | R | Included | 0.9 |
| 49 | I think Teachers try to improve their teaching in the light of student feedback | R | R | R | R | R | R | R | R | R | R | R | R |  |  | R |  | R | R | R | R | Included | 0.9 |

R= Relevant, NR= Not Relevant, E= expert, I-CVI= Content validity index

**APPENDIX IV: Delphi round Two**

| S. No | Items | E1 | E2 | E3 | E4 | E5 | E6 | E7 | E8 | E9 | E10 | E11 | E12 | Comments | CVR |
| --- | --- | --- | --- | --- | --- | --- | --- | --- | --- | --- | --- | --- | --- | --- | --- |
| 1 | Teachers encourage and promote group activities and interactive discussions. | E | E | E | E | E | E | E | NE | E | E | NE | NE | Removed | 0.5 |
| 2 | Teachers are well-organized and follow the schedule | E | E | E | E | E | NE | E | E | E | E | E | E | Included | 0.84 |
| 3 | The teachers are well-prepared for every session | E | E | E | E | E | E | NE | E | E | E | E | E | Included | 0.84 |
| 4 | Students are the focus of attention in every learning environment | E | E | E | NE | E | E | E | E | E | NE | E | E | Revised | 0.67 |
| 5 | Teachers have good time management skills | NE | NE | E | E | E | NE | E | NE | E | E | E | E | Removed | 0.5 |
| 6 | Learning objectives are clearly mentioned for each session. | E | E | E | E | E | E | E | NE | E | E | E | E | Included | 0.84 |
| 9 | Teachers use metacognitive strategies (clues, hints, schemas, examples, questions) during the course delivery | E | E | E | NE | E | E | E | E | E | E | NE | E | Revised | 0.67 |
| 10 | Teachers have effective communication skills | E | E | E | NE | E | E | E | E | E | E | E | E | Included | 0.84 |
| 11 | Teachers cover the content in appropriate time during the session | NE | NE | E | E | E | E | E | NE | E | E | NE | E | Removed | 0.5 |
| 12 | Teachers value my feedback on course and teaching activities | E | E | E | NE | E | E | E | E | E | E | E | E | Revised | 0.67 |
| 13 | Teachers encourage me to be active and lifelong learners. | E | E | E | NE | E | E | E | E | NE | E | E | E | Revised | 0.67 |
| 14 | Teachers provide me essential guidance suitable for my learning | E | E | E | E | E | E | NE | E | E | E | E | E | Included | 0.84 |
| 15 | Teachers provide me essential learning resources after the session. | E | E | E | E | E | E | E | E | E | E | E | E | Revised | 0.75 |
| 16 | Teachers built new knowledge on my prior knowledge | E | E | E | NE | E | E | E | E | E | NE | E | E | Revised | 0.67 |
| 17 | Teachers improve my knowledge and skills with their teaching. | NE | E | E | E | E | E | E | NE | E | E | E | E | Revised | 0.67 |
| 18 | Teachers cover the content in appropriate time during the session | E | E | E | E | E | NE | E | E | E | E | NE | E | Revised | 0.67 |
| 19 | I am offered incentives like scholarships if i perform well | E | E | NE | E | E | E | E | NE | NE | E | E | E | Removed | 0.5 |
| 20 | I am never mocked or ridiculed. | E | NE | E | E | E | E | E | E | E | E | NE | NE | Removed | 0.5 |
| 21 | Teachers have optimal teaching skills | NE | NE | E | E | E | E | E | E | E | E | E | NE | Removed | 0.5 |
| 22 | Teachers’ academic ability and qualification is up-to the mark | E | E | E | E | NE | E | E | NE | E | E | E | NE | Removed | 0.5 |
| 23 | Teachers have command over their subject/discipline | E | E | NE | E | E | E | E | NE | E | E | E | E | Revised | 0.67 |
| 24 | Teachers have some degree in medical education, in addition to major speciality. | E | E | NE | E | E | E | E | NE | E | E | NE | E | Removed | 0.5 |
| 25 | Teachers are well equipped with E teaching and assessment techniques. | E | E | NE | E | E | E | E | E | E | E | E | E | Included | 0.84 |
| 26 | Teachers provide me with timely and constructive feedback on my learning | E | E | E | E | E | E | NE | E | E | E | E | NE | Revised | 0.67 |
| 27 | Teachers use multiple and innovative teaching strategies | NE | E | E | E | E | E | E | E | E | E | E | E | Included | 0.84 |
| 28 | Teachers demonstrate Mentorship skills in teaching | E | NE | E | E | E | E | E | NE | E | E | E | NE | Removed | 0.5 |
| 29 | Teachers helped me in applying theoretical knowledge into practice. | E | E | E | E | E | E | E | E | E | E | E | E | Included | 1.0 |
| 30 | Tasks and activities assigned to me corresponded with my level of learning. | NE | E | E | E | E | E | E | E | E | E | E | NE | Revised | 0.67 |
| 31 | Teachers provide me a welcoming and friendly learning environment | E | E | NE | E | E | E | E | E | E | E | E | E | Included | 0.84 |
| 32 | I am involved in decision making processeS | E | E | NE | E | E | E | E | E | E | E | NE | NE | Removed | 0.5 |
| 33 | Teachers Encourage Peer Assisted Learning | NE | E | E | E | E | E | E | E | E | E | NE | NE | Removed | 0.5 |
| 34 | Teachers promote critical thinking rather than rote memorization | E | E | E | NE | E | E | E | E | E | E | E | E | Included | 0.84 |
| 35 | Teachers have an unbiased approach towards me. | NE | E | E | E | E | E | E | E | E | E | E | NE | Revised | 0.67 |
| 36 | . Teachers mock and ridicule the students | E | E | E | E | NE | E | E | E | E | E | E | NE | Revised | 0.67 |
| 37 | Teachers are nonjudgmental towards me | E | E | E | NE | E | E | E | E | E | E | NE | NE | Removed | 0.5 |
| 38 | . I feel that Teachers are, more interested in finishing the course, instead of learning of students | E | E | NE | E | E | E | E | E | E | NE | E | E | Revised | 0.67 |
| 39 | I think Teachers try to improve their teaching in the light of student feedback | E | NE | E | E | E | NE | E | E | E | E | NE | E | Removed | 0.5 |

E= Essential, NE= Non-Essential, CVR=Content Validity Ratio

**APPENDIX V: Results of Cognitive Interviews**

| **S. N** | **Themes** | **Items** | **Qualitative analysis** | **Codes** |
| --- | --- | --- | --- | --- |
| 1. | TEACHING PRACTICES | Teachers are well organized and follow the schedule | The statement was clear to the respondents” Teachers should be able to regulate their teaching activities as per their curriculum and teaching schedule.” (SR P2)  good” (SR P2). | No change |
| 2. |  | Teachers are well prepared for every session | “I agree that preparedness of the teacher is extremely important for any instructional method” (SR P4) | No Change |
| 3. |  | Learning objectives are clearly mentioned for each session | “I agree that teacher should always emphasized on learning objectives-oriented teaching as it gives a sense of direction to both the students as well as the teachers. (SR P3). | No change |
| 4. |  | Teachers use metacognitive strategies (clues, hints, schemas, examples, questions) during the course delivery | “It’s important to cater metacognition in this questionnaire” (SR P5) | No change |
| 5. |  | Teachers have effective communication skills | I totally agree that “clear communication is extremely important for good teacher/student relationship and for effective learning: (SR P1). | No change |
| 6. |  | Students are the center of attention in every learning environment | “It is important point, but It can be rephrased slightly, as in this case our focus is on microlearning environment, so students are the center of attention in microlearning environment” (SR P7). | Rephrase |
| 7. |  | Teachers value my feedback on course and teaching activities | student feedback is the cornerstone to steer teaching the statement can be made clearer by saying” Teachers try to improve their teaching in the light of student feedback” it is important point but should (SR P6). | Rephrase |
| 8. |  | Teachers encourage me to be an active and lifelong learner | The eventual goal of teaching should be to make active and lifelong learners. Therefore, it should be the priority of all teachers as well as this questionnaire (SR P9). | No change |
| 9. |  | Teachers provide me appropriate guidance required for my learning | Without appropriate guidance, students might struggle but can be improved further by using word “appropriate” instead of “essential” (CS P5). | No change |
| 10. |  | Teachers guide me about learning resources for every session | Adding “required “before “for every session” will be more appropriate | Rephrase |
| 11. |  | Teachers built new knowledge on my prior knowledge | I agree that schema formation should be the priority of every teacher and building onto prior knowledge is just that but should be slightly rephrased as  Teachers help me build new knowledge on my prior knowledge | Rephrase |
| 12. | LEARNERS SUPPORT | Teachers improve my knowledge and skills with their teaching. | Adding attitude will make the statement more comprehensive | Rephrase |
| 13. |  | Teachers provide me with constructive and timely feedback on my learning | Participants agreed with the point.” constructive feedback drives learning and skillset. great that you addressed it” | No change |
| 14. |  | Tasks and activities assigned to me corresponded with my level of learning | Convert the sentence from past to present tense | Rephrase |
| 15. | COMPETENCE IN TEACHING | Teachers, being positive role models,  are a source of inspiration for me | Only role models are enough don’t write “positive” | Rephrase |
| 16. |  | Teachers have command over their subject/discipline | Experts agreed on this point. | No change |
| 17. |  | Teachers help me in applying theoretical knowledge into practice | Convert the sentence from past to present tense). | Rephrase |
| 18. |  | Teachers are well equipped with online teaching and assessment techniques | e-learning is the way forward but rephrase it as “e learning and teaching techniques’ | Rephrase |
| 18. | PROGRESSIVE FACULTY | Teachers use multiple and innovative teaching methods | Experts agreed that no change is required | No change |
| 19. |  | Teachers emphasize on critical thinking rather than rote memorization | “This statement is crystal clear in its meaning and to be retained as such “ | No change |
| 20. |  | Teachers use welcoming learning environment | Teachers provide me with friendly learning environment. | Rephrase |
| 21 | TEACHING ENVIRONMENTS | Teachers have an unbiased approach towards all the students | This statement was deemed important by all participants | No change |
| 22 |  | Teachers mock and ridicule the students | This Statement was jointly agreed upon,”. Good that you have added a negative item as well “. | No change |
| 23 |  | I feel that Teachers are, more interested in finishing the course, instead of learning of students. | Statement should be rephrased as”. Teachers are interested in completing the course instead of clarification of concepts” for better understanding. | Rephrase |

**APPENDIX VI: QUESTIONAIRE:**

Faculty name:

Institution:

Qualification:

Academic Position at institute:

1. Professor
2. Associate professor
3. Assistant Professor
4. Lecturer
5. Any other please specify__________________________________________

Subject of teaching: ____________________________________________________

Teaching experience as a medical teacher (in years): _________________________

| SR NO | DOMAINS | ITEMS | NR/NC | SR/SC | QR/QC | HR/HC |
| --- | --- | --- | --- | --- | --- | --- |
| 1 | TEACHING PRACTICES | Teachers Are Well Organized and Follow the Schedule? |  |  |  |  |
| 2 |  | Teachers Are Well Prepared for Every Session? |  |  |  |  |
| 3 |  | Learning Objectives Are Clearly Mentioned for Each Session? |  |  |  |  |
| 4 |  | Teachers Use Metacognitive Strategies (Clues, Hints, Schemas, Examples, Questions) During the Course Delivery |  |  |  |  |
| 5 |  | Teachers Have Effective Communication Skills? |  |  |  |  |
| 6 | LEARNERS SUPPORT | Students Are the Center of Attention in Every Learning Environment? |  |  |  |  |
| 7 |  | Teachers Value My Feedback on Course and Teaching Activities? |  |  |  |  |
| 8 |  | Teachers Encourage Me to Be an Active and Lifelong Learner? |  |  |  |  |
| 9 |  | Teachers Provide Me Appropriate Guidance for My Learning? |  |  |  |  |
| 10 |  | Teachers Guide Me About Learning Resources for Every Session? |  |  |  |  |
| 11 |  | Teachers Help Me Build New Knowledge on My Prior Knowledge? |  |  |  |  |
| 12 |  | Teachers Improve My Knowledge, Skill and Attitude with Their Teaching? |  |  |  |  |
| 13 |  | Teachers Provide Me with Timely and Constructive Feedback on My Learning? |  |  |  |  |
| 14 |  | Tasks And Activities Assigned to Me Corresponds with My Level of Learning? |  |  |  |  |
| 15 | COMPETENCE IN TEACHING | Teachers, Being Role Models, Are Source of Inspiration for Me? |  |  |  |  |
| 16 |  | Teachers Have Command Over Their Subject/ Discipline? |  |  |  |  |
| 17 |  | Teachers Help Me in Applying Theoretical Knowledge into Practice |  |  |  |  |
| 18 | PROGRESSIVE FACULTY | Teachers Are Well Equipped with Online Teaching and Assessment Techniques? |  |  |  |  |
| 19 |  | Teachers Use Multiple and Innovative Teaching Methods? |  |  |  |  |
| 20 |  | Teachers Emphasize on Critical Thinking Rather Than Rote Memorization? |  |  |  |  |
| 21 | TEACHING ENVIRONMENTS | Teachers EncourageFriendly Learning Environment? |  |  |  |  |
| 22 |  | Teachers Have an Unbiased Approach Towards All Students? |  |  |  |  |
| 23 |  | Teachers Mock and Ridicule the Students |  |  |  |  |
